# Expanding the phenotypic spectrum of mutations in *LRP2*: a novel candidate gene of non-syndromic familial comitant strabismus

**DOI:** 10.1186/s12967-021-03155-z

**Published:** 2021-12-06

**Authors:** Yue Wang, Xuejuan Chen, Tao Jiang, Yayun Gu, Xiaohan Zhang, Wenwen Yuan, Andi Zhao, Rui Li, Zijin Wang, Zhibin Hu, Hu Liu

**Affiliations:** 1 Department of Ophthalmology, The First Affiliated Hospital With Nanjing Medical University, 300 Guangzhou Rd, Nanjing, 210029 China; 2 Department of Epidemiology, Center for Global Health, School of Public Health, Nanjing Medical University, Nanjing, China; 3 State Key Laboratory of Reproductive Medicine, Nanjing Medical University, 101 Longmian Rd, NanjingNanjing, 211166 China; 4 Department of Ophthalmology, Wuxi Children’s Hospital, Wuxi, China

**Keywords:** Comitant strabismus, Mutation, Phenotype, Whole-exome sequencing, Linkage analysis

## Abstract

**Background:**

Comitant strabismus (CS) is a heterogeneous disorder that is a major contributing factor to unilateral childhood-onset visual impairment. Studies have confirmed that genetic factors play an important role in the development of CS. The aim of this study was to identify the genetic cause of non-syndromic familial CS.

**Methods:**

Fourteen unrelated CS families were recruited for the study. Twelve affected and 2 unaffected individuals from a large four-generation family (CS08) were selected to perform whole genome-wide linkage analysis. Parallel whole-exome sequencing (WES) was conducted in the same family (9 patients and 1 unaffected member) and 31 additional CS cases from 13 other unrelated families. Sanger sequencing was used to determine whether any of the remaining variants co-segregated with the disease phenotype in the corresponding family.

**Results:**

Based on linkage analysis, CS in family CS08 mapped to a novel region of 34.17 centimorgan (cM) on chromosome 2q22.3-2q32.1 between markers D2S151 and D2S364, with a maximum log odds (LOD) score of 3.54 (theta = 0) at D2S142. Parallel WES identified a heterozygous variant, *LRP2* c.335 A > G (p.Q112R), located in such a linkage interval that completely co-segregated with the disease in the family. Furthermore, another novel heterozygous variant (c.7274A > G, p.D2425G) in *LRP2* that co-segregated was detected in 2 additional affected individuals from another unrelated family by WES. Both variants are predicted to be damaging by PolyPhen-2, SIFT and MutationTaster, and were absent in 100 ethnically matched normal controls.

**Conclusion:**

*LRP2* is a novel candidate genetic cause of non-syndromic familial CS.

**Supplementary Information:**

The online version contains supplementary material available at 10.1186/s12967-021-03155-z.

## Introduction

Strabismus is clinically defined as a condition in any misalignment of the eyes in coordination, which is a major ocular abnormality in children and often accompanied by adverse effects on binocularity, stereopsis, and depth of perception. Epidemiological data indicate a prevalence of approximately 1–4% approximately in some populations [1, 2]. According to the change in the magnitude of misalignment in different gaze directions, strabismus can be subclassified as comitant strabismus (CS; constant in all directions) and incomitant strabismus (various). CS is the most common form of strabismus and a major contributing factor to unilateral childhood-onset visual impairment, especially amblyopia [2, 3]. In addition, strabismus affects normal binocular vision function even in the absence of amblyopia, subsequently affecting daily physiological and psychosocial performance unless successfully treated [4, 5].

CS is highly heterogeneous and influenced by genetic and environmental factors, though the pathogenesis remains unclear [6, 7]. Based on the inheritance pattern, numerous studies have been performed to investigate the genetic causation of CS using different methods. Considering the complex influencing background and high prevalence of this disease, two genome-wide association studies were recently carried out for strabismus, two variants in *TSPAN10* (rs6420484 and rs397693108) and a variant in *WRB* (rs2244352) were found to increase the susceptibility to strabismus [8, 9]. However, previous family, twin, and pedigree studies have confirmed that some CS families show an autosomal dominant (AD) or autosomal recessive (AR) pattern of inheritance [10–12]. Linkage analysis has also implicated several associated loci, with the most significant being chromosome 7p22.1 (STBMS1 locus, OMIM: 185100), transmitting in both AR and AD models [13, 14]. Moreover, two other susceptibility loci, 4q28.3 and 7q31.2, were recently identified in the Japanese population in association with the candidate genes *MGST2* and *WNT2* [15, 16]. In addition, variants of *AHI1* and *PAX3* have been detected by whole-exome sequencing (WES) in Chinese families, contributing to strabismus [17, 18]. Anyway, these studies indicate that CS may manifest as a rare and monogenic subtype.

In the current study, we recruited 14 unrelated non-syndromic CS-affected Chinese families, including a large four-generation family, CS08. WES and genome-wide linkage analysis were performed synchronously to identify a rare heterozygous variant, c.335 A > G (p.Q112R) in the *LRP2* gene, located in the corresponding linkage interval (2q22.3-2q32.1) and co-segregating with the disease in the family CS08. WES also detected another heterozygous variant (c.7274A > G, p.D2425G) in *LRP2* in two additional affected individuals from another unrelated CS family (CS06).

## Materials and methods

### Families and clinical examinations

**Fig. 1:**
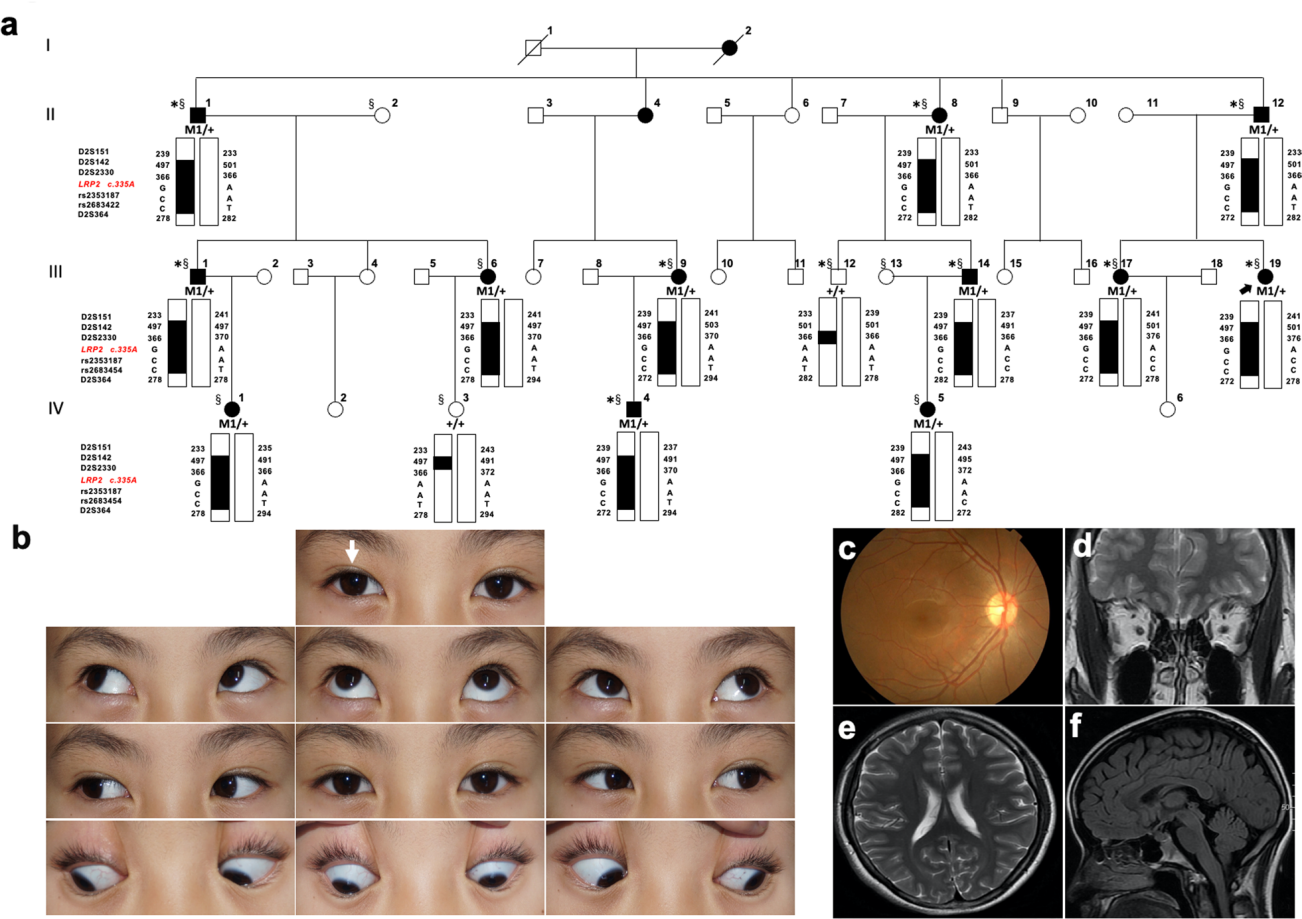
Pedigree of family CS08 and haplotype reconstruction for the mapped region on chromosome 2 and clinical evaluations of the proband in family CS08. **a** Affected and unaffected members are indicated by filled and open symbols respectively. The black arrow indicates the proband. Haplotypes for tested short tandem repeat (STR) markers and genotypes for *LRP2* c.335, rs2683454 and rs2683454, are given for all participants. Black bars represent the ancestral haplotype associated with the disease. *Individuals on whom WES was performed, ^§^Individuals on whom sanger sequencing was performed. Abbreviation: M1, mutation c.335A > G **b** Ocular positions and movements. White arrow, exotropia phenotype of right eye. **c** Fundus photograph of right eye. **d** Ocular MRI. **e–f** Brain MRI for patient III:19

Forty-seven non-syndromic CS-affected and 18 CS-unaffected siblings from 14 unrelated families (1 with esotropia and 13 with exotropia; Fig. 1a and 3a, and Additional file 2a-l), including a large four-generation family (CS08), were recruited and clinically followed-up at the First Affiliated Hospital with Nanjing Medical University. Written informed consent was obtained from the participants and the parents of each child for sample collection and genetic analysis, and this study was approved by the ethics committee of the First Affiliated Hospital with Nanjing Medical University (2019-SR-134) in accordance with the Declaration of Helsinki principles.

Before they received any treatment, routine ocular examinations were performed on available participants, including visual acuity, slit-lamp biomicroscopy, and funduscopic evaluations. Angles of deviation at a distance (5 m) and at near (0.3 m) with the cover/uncover test, alternate prism and cover test or Krimsky test (in young or uncooperative patients) were also carried out. Refractive errors were measured using an autorefractometer. Ocular and brain magnetic resonance imaging (MRI) was performed on the proband of family CS08. Renal function was investigated using urine and blood samples from the two probands of families CS08 and CS06. Data for the history of strabismus treatment, including prior surgery or patching, were obtained before the examination from available participants themselves or confirmed by telephone conversations.

Strabismus was defined if any tropia was present at a distance or near, with or without wearing spectacles, and classified according to the primary direction (esotropia, exotropia, vertical) of the tropia. Strabismus can be subclassified according to the change in magnitude of misalignment in different directions of gaze as CS (comitant) and incomitant strabismus (various). Strabismus is considered constant tropia if constant at both near and distance fixation; otherwise, it is considered intermittent tropia. To minimize the effect of environmental and syndromic factors, cases according to the following criteria were excluded [13]: (i) any secondary strabismus; (ii) any incomitant strabismus; (iii) individual with known CS risk factors such as prematurity (< 35 weeks of age), low birth weight (< 1.8 kg); (iv) strabismus caused by deprivation or myasthenia gravis.

Another 100 unrelated ethnically matched normal controls free of brain and ocular diseases were also recruited. Genomic DNA was isolated from peripheral venous blood (5 ml) using TIANamp Genomic DNA Kit (TIANGEN, Beijing, China).

### Whole genome-wide linkage analysis

Whole genome-wide linkage screening was performed on the largest family CS08, including 12 patients (Fig. 1a; II:1, II:8, II:12, III:1, III:6, III:9, III:14, III:17, III:19, IV:1, IV:4 and IV:5) and two unaffected members (Fig. 1a; III:12 and IV:3). Moreover, 366 microsatellite markers and 3 single nucleotide polymorphisms (SNPs) spanning the entire human genome with an interval of approximately 10 cM (Weber set 6.0) were amplified by polymerase chain reaction (PCR) using primers labelled with Fam (Additional file 1). The PCR products were appropriately pooled according to allele size and labelling, mixed with GeneScanTM—500 Liz Size Standard (Applied Biosystems, Foster City, CA), denatured, loaded onto 6% standard denaturing polyacrylamide gels, and processed using an ABI 3730xl Analyzer (Applied Biosystems) for fluorescent detection. The pedigree displayed male-to-male transmission (Fig. 1a; II:1 and III:1) of the disease and almost equal numbers of affected males and females, indicating an AD pattern of inheritance (Fig. 1a). The multipoint LOD score was calculated using an AD inheritance model with 0.0001 and 0.01 disease allele frequencies and a penetrance range from 80 to 100%, respectively. Genotyping data were collected and analysed using the Genemapper 4.1 software package (Applied Biosystems). Multipoint linkage analysis was performed with the MERLIN program (http://www.sph.umich.edu/csg/abecasis/Merlin/index.html). Family and haplotype data were generated using Cyrillic, Version 2.1 program.

### Whole exome sequencing and Sanger sequencing

Parallel WES was carried out using genomic DNA from 9 patients (Fig. 1a; excluding III:6, IV:1, and IV:5 compared with linkage screening) and 1 unaffected member (III:12) of family CS08. WES was performed on genomic DNA from 31 additional patients from 13 unrelated families (14 males and 17 females, Additional file 2). WES was performed with a SureSelect Human All Exon 50 Mb Kit (Agilent Technologies, Santa Clara, CA) and sequenced on the HiSeq 2000 platform (Illumina, San Diego, CA). CASAVA v1.8.2 was used to convert Illumina BCL files to FASTQ files. Low-quality bases and adapters were filtered with Trimmomatic version 0.32 [19]. Then, sequence reads were mapped to the human reference sequence (GRCh37) with default parameters by Burrows-Wheeler Aligner (BWA-MEM v0.7.15-r1140) [20]. Single nucleotide variants (SNVs) and small insertions and deletions (INDELs) were called using GATK Best Practice pipelines [21]. Base-quality score recalibration and local alignment around INDELs were refined by the GATK suite version 3.5.0. We used snpEff (v4.3–3) to annotate variants with population frequency, phylogenetic conservation scores, gene regions, and exonic functions, after which all annotated variants were loaded into the GEMINI (v0.19.1) [22]. Sanger sequencing and intrafamilial co-segregation analysis on the variants shared among all patients and absent in unaffected members.

### In silico analyses

Pathogenicity prediction was performed by using three online mutational pathogenicity evaluation software programs: SIFT (http://sift.jcvi.org/); PolyPhen-2 (http://genetics.bwh.harvard.edu/pph2/); and Mutationtaster (http://www.mutationtaster.org/). Evolutionary conservation of the mutated residue was analysed with Ensemble Genome Server database BLAST / BLAT Tools by aligning the protein sequence of human LRP2 with sequences of the following orthologous proteins: *Pan troglodytes* (XP_515882.2), *Macaca mulatta* (XP_001104179.2), *Bos Taurus* (XP_002685354.2), *Mus musculus* (NP_001074557.1), *Rattus norvegicus* (NP_110454.1), *Gallus gallus* (XP_004942820.1), and *Danio rerio* (NP_001181916.1). Crystal structural models of wild-type and mutant LRP2 were constructed using the SWISS-MODEL online server. Predicted structures were displayed with PyMol software (version 1.5).

## Results

### Characterizations of the four-generation family CS08

The pedigree of the family CS08 is depicted in Fig. 1a. All of the affected individuals exhibited the strabismus phenotype with comitant exotropia. A total of 12 patients and two unaffected individuals in this family were recruited, comprising six males and eight females. The proband (III:19) was a 22-year-old female with intermittent exotropia, which occurred at approximately 5 years of age. The amount of tropia before surgery displayed—30 PD (near), and—25 PD (far) with best-corrected visual acuity (BCVA) was 1.0 in both eyes (Fig. 1b and Table 1). The refractive error status was slight astigmatism in the left eye (-1.00 D). No abnormality was found by slit-lamp biomicroscopy and funduscopic evaluations of the two eyes (Fig. 1c). Both ocular and brain MRI appeared normal (Fig. 1d–f). The proband denied any renal symptoms, and normal results were confirmed by renal function detection. In addition to the patient, III:6 presented constant exotropia in the right eye; the remaining 11 patients presented different degrees of intermittent exotropia. All patients were born via normal pregnancy and delivery. None of the patients, except for the proband, had a prior history of treatment. Moreover, no other syndromic feature was observed in any of the individuals. The ocular clinical data of this family are summarized in Table 1.

**Table 1:** Clinical features of included participants in CS08

Patient ID	Age (yrs)	Gender	Diagnosis	Spherical power (D)		BCVA	
				**O.D**	**O.S**	**O.D**	**O.S**
II:1	63	M	IXT	– 0.50	– 0.50	0.6	0.6
II:8	58	F	IXT	0	– 0.50	1.0	1.0
II:12	55	M	IXT	0	– 0.25	1.0	0.6
III:1	42	M	IXT	– 1.00	– 0.75	1.0	1.0
III:6	33	F	CXT	– 3.00	– 2.50	0.6	1.0
III:9	39	F	IXT	– 1.25	– 1.00	1.0	0.8
III:12	36	M	NOR	0	+ 0.25	1.0	1.0
III:14	34	M	IXT	– 3.35	– 3.50	1.0	1.0
III:17	26	F	IXT	– 3.25	– 3.50	1.0	1.0
III:19	22	F	IXT	0	+ 1.00	1.0	1.0
IV:1	19	F	IXT	– 3.00	– 3.00	1.0	1.0
IV:3	18	F	NOR	– 2.00	– 1.50	0.8	1.0
IV:4	14	M	IXT	– 5.00	– 5.00	1.0	1.0
IV:5	9	F	IXT	– 1.25	– 1.00	1.0	1.0

D, diopter; BCVA, best corrected visual acuity; O.D., right eye; O.S.. left eye; IOL, intraocular lens; IXT, intermittent exotropia; CXT, constant exotropia

### Linkage analysis of initially located pathogenic genes on chromosome 2

**Fig. 2:**
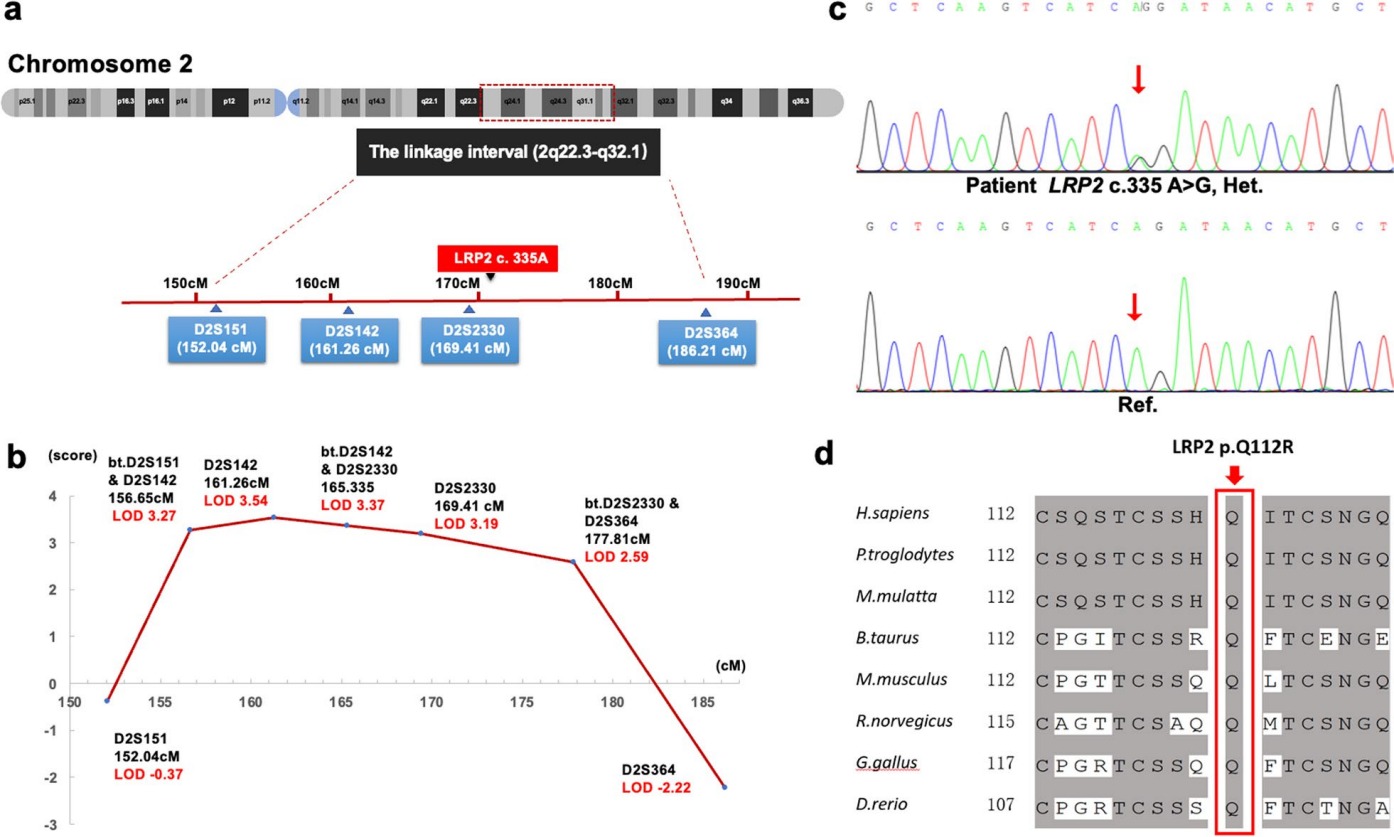
Multipoint linkage analysis results and analysis of mutation c.335 A > G (p.Q112R) in *LPR2*. **a** The locus for chromosomal region 2q22.3-2q32.1. Relative order of genotyped microsatellite markers is shown at the bottom next to an ideogram of chromosome 2. **b** Results from multipoint linkage analysis and genetic locations for the markers genotyped. The horizontal axis represents position of Chromosome 2. The vertical axis represents LOD score. bt., between. **c** Sanger sequencing showing heterozygous c.335 A > G mutation in III:19 (patient) and III:12 (unaffected) respectively. Reference sequences are given at the bottom. Abbreviation: Het., heterozygous; Ref., reference. **d** Conservation analysis of residue p.Q112 (boxed) of LRP2 across eight species

To determine the genetic cause of non-syndromic comitant exotropia in family CS08, we performed the whole genome-wide linkage scan using genomic DNA from family CS08 (12 patients and 2 unaffected members). We calculated the multipoint LOD score for family CS08 under 0.0001 and 0.01 disease allele frequencies and penetrance varying from 80 to 100%. Multipoint linkage analysis identified a 34.17 centimorgan (cM) candidate region co-segregating with the disease on chromosome 2q22.3-2q32.1 using the dominant model. The critical interval is flanked by markers D2S151 and D2S364, with a maximum LOD score of 3.54 (theta = 0) at D2S142 under a model in which the disease allele frequency was 0.01 and penetrance is 100% (Fig. 2a, b, Additional file 3). Haplotype construction is illustrated in Fig. 1a.

### WES and Sanger sequencing identified a candidate gene

Parallel WES was performed on genomic DNA from 9 affected and 1 unaffected member of the CS08 family pedigree (Fig. 1a). Initially, a total of 112,804 variants were detected; each sample had a mean depth of 188 × , with at least 5 × coverage over 98.98% of the reference genome. As shown in Additional file 4, after bioinformatics analysis and filtering, only one heterozygous missense variant *LRP2* c.335 A > G, (p.Q112R) remained. This missense mutation is predicted to be disease-causing by prediction tools (SIFT, PolyPhen-2, and MutationTaster) (Table 2). This variant is located inside the linkage interval on 2q22.3-2q32.1, with complete co-segregation with the disease in this family based on Sanger sequencing, which was conducted for all 14 CS08 members (Fig. 1a, 2c). Furthermore, Sanger sequencing of the same site was performed in 100 normal controls, with no positive result. Therefore, we believe that *LRP2* is likely to be the causative gene of CS in this family.

**Table 2:** *LRP2* mutations Identified in the CS06 and CS08

Family ID	Variation				Exon	Bioinformatics Analysis			Conservation Analysis	Frequency in Databases				
	Nucleotide*	**Amino Acid**	**Type**	**Status**		**SIFT**	**PolyPhen-2**	**Mutationtaster**		**1000G (Al l)**	**1000G (EAS)**	**gnomAD (All)**	**gnomAD (EAS)**	**TOPMed**
CS08	c.335A > G	p. Q112R	M	Het	4	Damaging /0.004	Prob /0.955	Disease causing	Conserved	None	None	0.00002389	0.0003262	None
CS06	c.7274A > G	p. D2425G	M	Het	39	Damaging /0.003	Prob /0.819	Disease causing	Conserved	None	None	None	None	None

M, Missense; Het, heterozygous; Prob, probably damaging; HGMD, the Human Gene Mutation Database; ExAC, Exome Aggregation Consortium; 1000G, 1000 Genomes Project; EAS, East Asian; gnomAD, The Genome Aggregation Database; TOPMed, Trans-Omics for Precision Medicine Program

^*^Sequence data from this article have been deposited with the GenBank Data Libraries under Accession NM_004525

### Detection of *LRP2* mutations in 13 additional families with CS

**Fig. 3:**
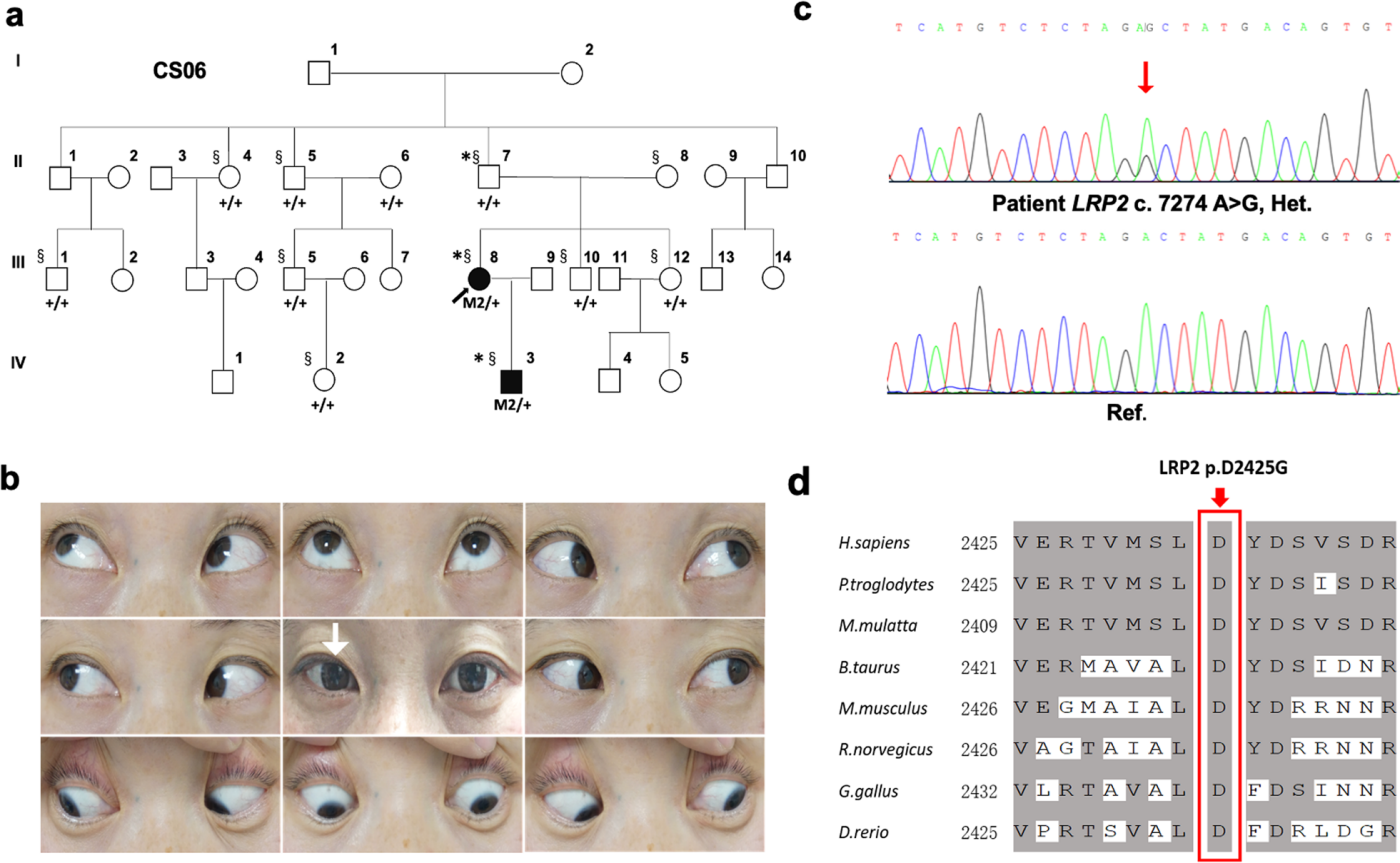
Pedigree of family CS06, clinical evaluations of the proband, and analysis of mutation c.7274A > G (p.D2425G) in *LPR2.* **a** Pedigree of family CS06. *Individuals on whom WES was performed, ^§^Individuals on whom sanger sequencing was performed. Abbreviation: M2, mutation c.7274A > G. **b** Ocular positions and movements for patient IV:3. White arrow, esotropia phenotype of right eye. **c** Sanger sequencing showing heterozygous c.7274A > G mutation in IV:3 (patient) and III:5 (unaffected) respectively. Reference sequences are given at the bottom. Abbreviation: Het., heterozygous; Ref., reference. **d** Conservation analysis of residue p.D2425 (boxed) of LRP2 across eight species

To assess the possibility of the genetic contribution of the *LRP2*, we further performed WES on 31 affected members of the other 13 unrelated CS families. An additional heterozygous variant (c.7274A > G, p.D2425G) in *LRP2* was detected in the proband of family CS06 (Fig. 3a). This variant was confirmed to co-segregate with the phenotype by Sanger sequencing, which was performed on all family members available (II:4, II:5, II:7, II:8, III:1, III:5, III:8, III:10, III:12, IV:2, IV:3). The proband III:8 was affected by comitant esotropia, which occurred at approximately 2 years of age. The amount of tropia displayed + 25 PD (near) and + 15 PD (distant); BCVA was 1.0 in both eyes (Fig. 3b). Refractive error status was moderate myopia (-2.75 D) in her left eye. Her son (IV:3), a 4-year-old boy with comitant esotropia, showed tropia of + 50 PD (near) and + 45 PD (distant). Sanger sequencing revealed a de novo mutation transmitted from his mother (III:8) that was present in his grandparents (Fig. 3a, c).

### Pathogenic analysis

Both heterozygous variants are absent or extremely rare in public databases and predicted to be disease-causing by SIFT, PolyPhen-2, and MutationTaster (Table 2). Furthermore, both of the missense variants (Q112R and D2425G) are highly conserved across different species (Fig. 2d, 3d), which supports the pathogenicity of *LRP2* variants causing CS.

**Fig. 4:**
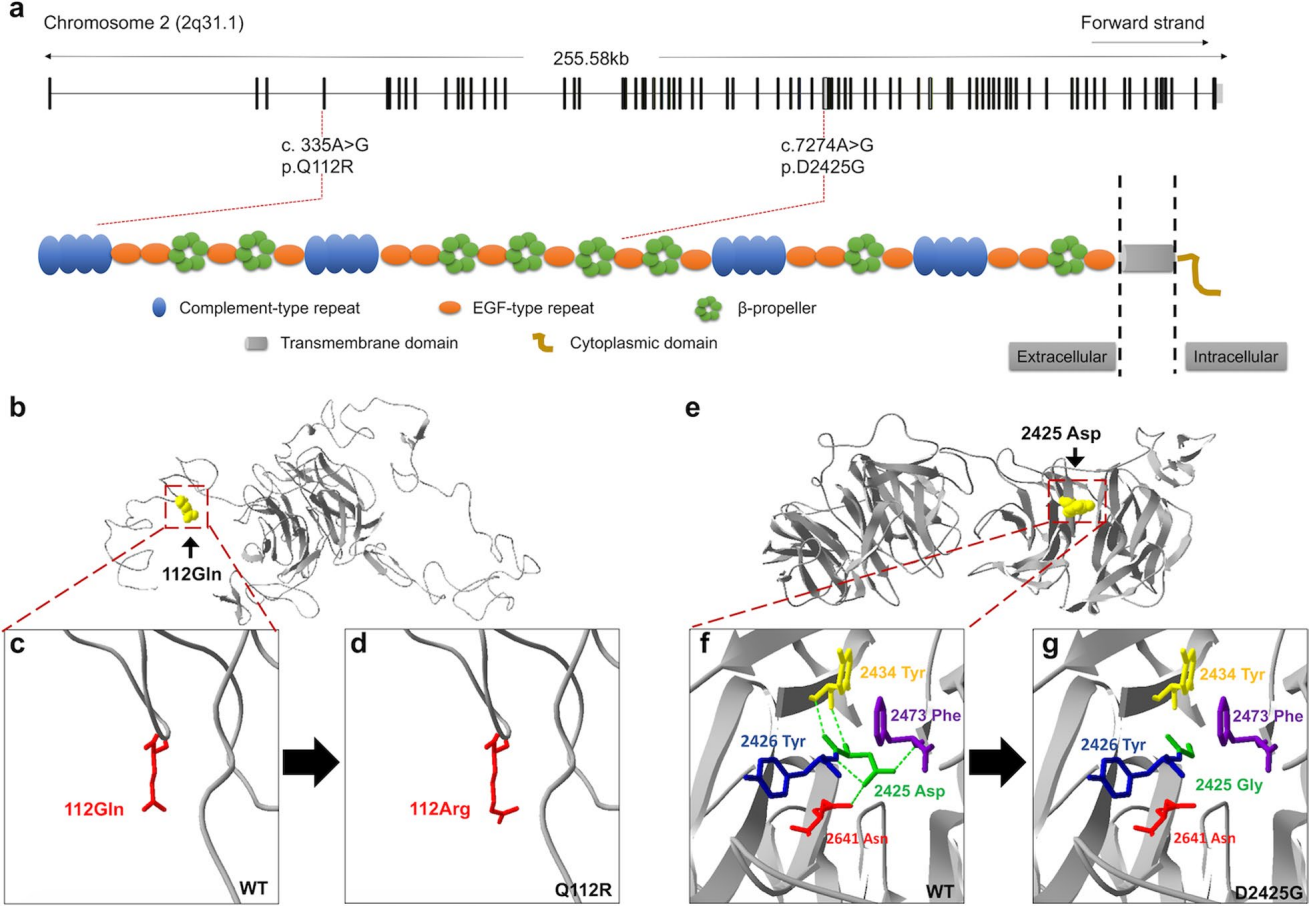
Schematic structural of LRP2/megalin and predicted crystal structural models of the wild-type and mutant. **a** Megalin is composed of a large extracellular domain, a single transmembrane domain, and a short cytoplasmic domain. The extracellular domain harbors four cysteine-rich complement-type ligand binding repeats, which are separated from each other by β-propellers and EGF-like repeats. **b-d** Crystal structures of wild-type human LRP2 and mutant human LRP2 carrying p.Q112R. **c** The mutation spot is highlighted red. D, The 112 residue glutamine is replaced by arginine. **e–g** Crystal structures of wild-type and mutant human LRP2 carrying p.D2425G. **e** The mutated residue is indicated in green. Amino acids interacted with residue 2425, including Tyr2426, Tyr2434, Phe2473, and Asn2641, are indicated. g The hydrogen bond between residue 2425 and Tyr2434, Tyr2426, Phe2473, as well Asn2641 are eliminated upon the change from wild-type aspartic to mutant glycine

The structural organization of LRP2 is shown in Fig. 4a; it is composed of complement-type repeats (CRs), epidermal growth factor (EGF)-like repeats, and β-propellers. Mutation Q112R is located in a CR, and D2425G is located between two β-propellers; both mutations are located in the extracellular domain. We performed crystal structural modelling for the mutant LRP2 using SWISS-MODEL to predict the pathogenic effect caused by two mutations (Fig. 4b-g) and found that the hydrogen bonds between residues 2425 and Tyr2434, Tyr2426, Phe2473, as well Asn2641 are eliminated upon the change from a wild-type aspartic to mutant glycine. It is likely that the mutation p.D2425G affects the folding and relevant biological process of LRP2.

## Discussion

In this study, we mapped non-syndromic CS in a four-generation family to a linkage interval on chromosome 2q22.3-2q32.1 (34.17 cM), with a maximum LOD score of 3.54. Furthermore, a rare heterozygous variant in *LRP2* (c.335A > G, p.Q112R) located in the corresponding linkage interval that completely co-segregates with the disease in the family was detected by WES. This missense variant *LRP2* c.335A > G exhibits a very low allelic frequency of 0.00002389 in gnomAD and was absent in other public databases (1000G and TOPmed, Table 2), indicating it was not a common benign polymorphism represented by these databases. WES also revealed another heterozygous variant of *LRP2* (c.7274A > G, p.D2425G) in 2 additional affected individuals from another unrelated family with CS. Both variants are highly conserved, absent or extremely rare in public databases; they were absent in 100 ethnically matched normal controls according to Sanger sequencing. These mutations are predicted to be damaging by PolyPhen-2, SIFT and Mutation Taster. These data indicated that mutations in *LRP2* are novel genetic causes of non-syndrome familial CS.

*LRP2*, located on chromosome 2q31.1, encodes a giant multiligand transmembrane receptor (600 kDa; also named megalin) of the low-density lipoprotein (LDL) receptor gene family [23, 24]. The structure of LRP2/megalin is depicted in Fig. 4a, consisting of a large extracellular domain, a single transmembrane domain, and a short cytoplasmic domain. The extracellular domain harbours four cysteine-rich complement-type ligand binding repeats, which are separated from each other by β-propellers and EGF-like repeats. The single transmembrane domain is connected to the intracellular segment, and the cytoplasmic tail is rich in multiple functional elements. LRP2 is highly expressed in epithelial cells in mammals, including the kidney, brain, eye, lung, and reproductive tissues. It binds many ligands associated with diverse signalling pathways, including Sonic Hedgehog (Shh), bone morphogenic protein (BMP), and retinoid trafficking, etc. [23–25]. Most *LRP2* mutations to date are associated with Donnai-Barrow syndrome (DBS), also known as facio-oculo-acoustico-renal syndrome, which is a rare autosomal-recessive and multi-system condition involving craniofacial features, ocular abnormalities, developmental delay, agenesis of the corpus callosum (ACC), intellectual disability, sensorineural hearing loss, and proteinuria [23, 26–28]. The universal ocular features of DBS are hypertelorism and high myopia; others, such as retinal detachment, iris coloboma, progressive visual loss, and optic nerve hypoplasia, have been mentioned in several cases. In addition, mutations in *LRP2* may contribute to Stickler syndrome and autosomal recessive non-syndromic intellectual disability [29, 30]. Interestingly, few cases of DBS, Stickler syndrome, and non-syndromic intellectual disability have been accompanied by strabismus, both exotropia and esotropia [28–30]. Nevertheless, it is unknown whether the strabismus exhibited in these patients is a primary phenotype caused by *LRP2* deficiency or merely a secondary change from abnormal development and function of the brain and/or ocular organs.

In our study, none of the patients from families CS08 and CS06 showed ocular symptoms or multi-system features other than CS. Despite intrafamilial phenotypic variability, high myopia was often observed in these DBS patients, ranging from -12.5 to -22.0 D, and was accompanied by large eyes. However, the refractive error state of proband III:19 was only slight astigmatism in the left eye (-1.00 D); ocular features of DBS were also absent in all other patients from family CS08. In the other family, CS06, both patient III:8 and her son IV:3 had a normal refractive state, with moderate myopia in the left eye of III:8. None of the members of the two families had anisometropia. In addition, characteristic craniofacial features and bulbophthalmia were absent in the two families. The results of routine renal function detection revealed a lack of proteinuria in both probands. Based on the above, the CS08 and CS06 patients could not be classified as having DBS. Therefore, we assume that *LRP2* is a possible genetic contributor to primary strabismus; the two *LRP2* mutations in this study are associated with an independent familial CS phenotype.

Multiple *LRP2*-deficient animal models exhibit frequent ophthalmic eye enlargement and high myopia, comparable to the phenotypes in DBS patients, involving both homozygous and compound heterozygous states [31–35]. Interestingly, adult heterozygous variant fish showed normal eye sizes and slight hyperopia [31–35], similar to the heterozygous carrier cases [28–30]. These data may explain the possible reason for the absence of eye enlargement, high myopia, and systemic features in our patients. In additional, neither strabismus was observed in these *LRP2*-deficient models (mouse and zebrafish). Despite the various phenotypes caused by mutations in *LRP2*, the choice of model animals (i.e., zebrafish and mice) in the above studies is also another possible explanation for the lack or limited binocular vision. In some cases, fish displayed asymmetry in the level of enlargement of their two eyes. However, anisometropia and bulbophthalmia were absent in the participants with strabismus in our study. Therefore, it is unlikely that ocular misalignment occurred subsequent to those abnormalities.

Overall, the pathogenesis of CS remains unclear, and various hypotheses have been proposed. Among these, ocular misalignment being likely caused by a disruption of binocular vision during the early critical period for development is universal [36, 37]. Normal binocular vision is dependent on interhemispheric connections, which are achieved by the corpus callosum (CC), the major fibre bundle in the mammalian brain [37]. In particular, the visual callosa connect the homologous regions of the visual cortex and combine the two halves of the visual field [37]. A number of observations and experiments in humans and cats have shown alterations in interhemispheric connections via the CC in strabismic eyes [38, 39]. It is worth noting that CC is a major forebrain-derived structure of white matter in the brain; mutations in *LRP2* lead to ACC (variable) in humans [23, 26–30]. Reported data confirmed that during forebrain development, LRP2 is the main auxiliary receptor of the SHH signalling pathway, and defect in this protein causes SHH/Patch1/LRP2 complex failure formation, thus affecting downstream signalling pathway activation [24, 25, 40]. Knockout of the *LRP2* gene causes non-split deformity of the forebrain in mice [27, 31, 35]. Evidence from the above studies strengthens the idea that *LPR2* is likely to be an important participant in regulating the brain and eye movement network.

There are some limitations to our study. First, due to patient rejection of functional MRI and diffusion-based tensor, we could not confirm whether there was an alteration in interhemispheric connection via the CC that existed; nevertheless, the CC structure of the proband appeared normal by brain MRI. Second, besides of the family CS06 and CS08, neither candidate variant was detected in another 12 families, confirming the complex genetic trait of CS.

## Conclusions

In summary, based on genome-wide linkage analysis, WES, and pathogenic analysis, we mapped non-syndromic CS to a novel locus on chromosome 2q22.3-2q32.1 and identified the rare heterozygous variant c.335A > G (p.Q112R) and novel heterozygous variants c.7274A > G, (p.D2425G) in *LRP2*. To the best of our knowledge, our study is the first report that *LRP2* is a genetic cause of non-syndromic CS, expanding the phenotypic spectrum of *LRP2* mutations. Similar to other known genes associated with CS, *LRP2* variants can only explain a small subgroup of CS. Meanwhile, the pathogenicity of *LRP2* variants in non-syndromic CS and mechanisms linking *LRP2* and CS require further elucidation.

## Supplementary Information

**Additional file 1:** **Table S1.** Information of microsatellite markers used in the study.

**Additional file 2:** **Figure S1.** Pedigree of the other 12 families. A-E. CS01 – CS05. F. CS07. G-L. CS09 – CS14. *Individuals from whom blood samples were collected. ^§^Individuals on whom WES were performed.

**Additional file 3:** **Table S2.** Details of microsatellite markers with LOD score > 0.

**Additional file 4:** **Figure S2.** Flow chart of variant analyses.

## Data Availability

The datasets used and/or analyzed during the current study are available from the corresponding author on reasonable request.

## Abbreviations

CS: Comitant strabismus
WES: Whole-exome sequencing
cM: Centimorgan
LOD: Log odds
CS: Comitant strabismus
AD: Autosomal dominant
AR: Autosomal recessive
MRI: Magnetic resonance imaging
PCR: Polymerase chain reaction
SNVs: Single nucleotide variants
INDELs: Insertions and deletions
BCVA: Best-corrected visual acuity
CR: Complement-type repeats
EGF: Epidermal growth factor
LDL: Low-density lipoprotein
Shh: Sonic Hedgehog
DBS: Donnai-Barrow syndrome
ACC: Agenesis of the corpus callosum
D: Diopter
