# Supplementary material for: Expanding the phenotypic spectrum of mutations in LRP2: a novel candidate gene of non-syndromic familial comitant strabismus
Source: J Transl Med. 2021 Dec 6;19:495. doi: 10.1186/s12967-021-03155-z (PMC8647414; doi:10.1186/s12967-021-03155-z)
Supplement: Supplementary file 2 — Additional file 2: Figure S1. Pedigree of the other 12 families. A-E. CS01 – CS05. F. CS07. G-L. CS09 – CS14. *Individuals from whom blood samples were collected. §Individuals on whom WES were performed. [file 12967_2021_3155_MOESM2_ESM.docx]

**Additional file 2**

**
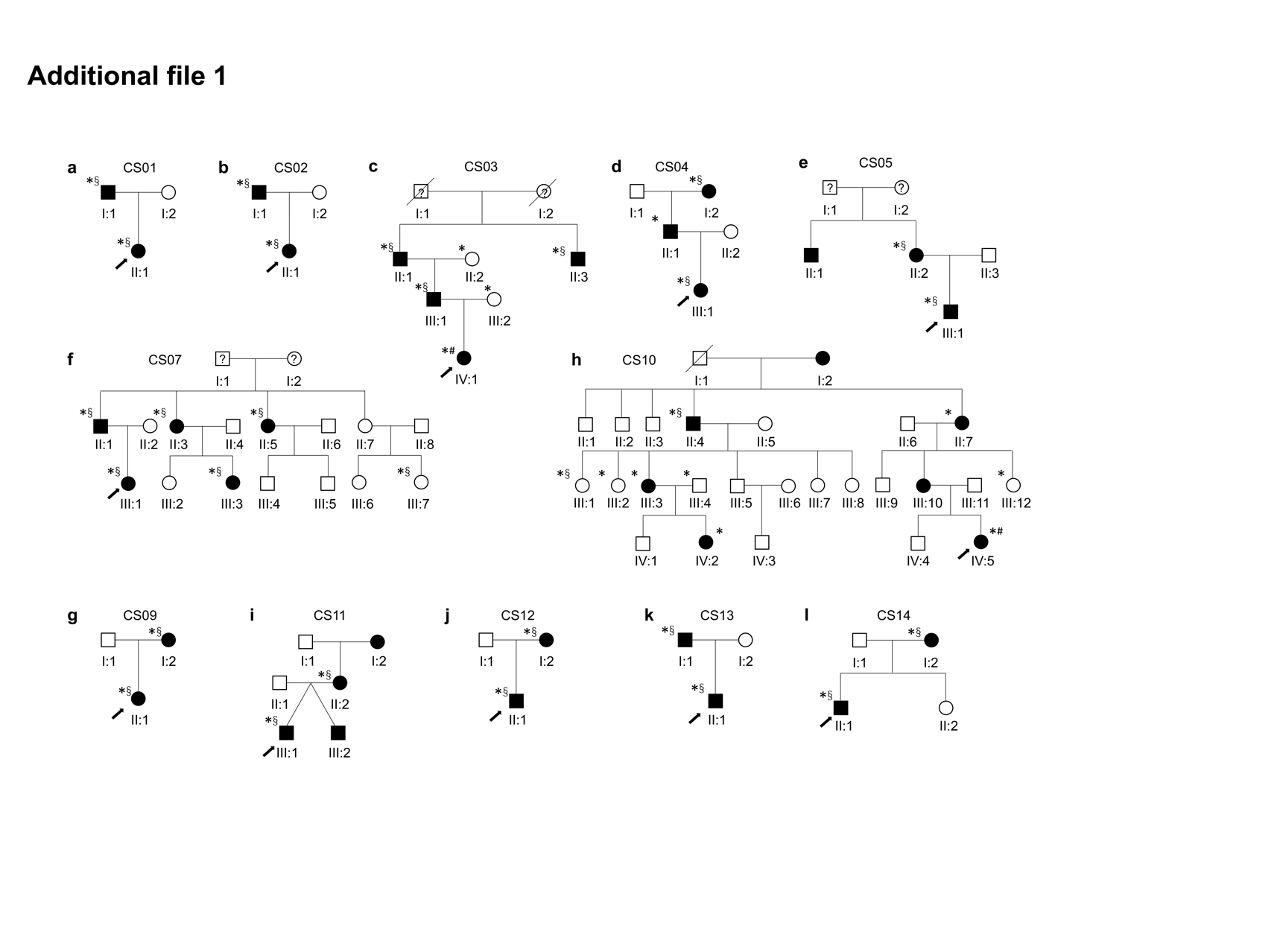
**

**Figure S1.** Pedigree of the other 12 families. A-E. CS01 – CS05. F. CS07. G-L. CS09 – CS14. *Individuals from whom blood samples were collected.

^§^Individuals on whom WES were performed.
