# Supplementary material for: Expanding the phenotypic spectrum of mutations in LRP2: a novel candidate gene of non-syndromic familial comitant strabismus
Source: J Transl Med. 2021 Dec 6;19:495. doi: 10.1186/s12967-021-03155-z (PMC8647414; doi:10.1186/s12967-021-03155-z)
Supplement: Supplementary file 3 — Additional file 3: Table S2. Details of microsatellite markers with LOD score > 0. [file 12967_2021_3155_MOESM3_ESM.docx]

**Additional file 3**

**TableS2.** Details of microsatellite markers with LOD score > 0.

| Chr | STR Marker | Genetic Marshfield (cM) | LOD Score under different penetrance | | | |
| --- | --- | --- | --- | --- | --- | --- |
|  |  |  | 80% | 90% | 99% | 100% |
| *Under 0.0001 disease allele frequency* | | | | | | |
| 2 | D2S337 | 80.69 | 0.93 | 0.77 | 0.28 | 0.13 |
| 2 | bt. D2S337 & D2S286 | 87.37 | 0.95 | 0.80 | 0.36 | 0.24 |
| 2 | bt. D2S112 & D2S151 | 146.83 | 0.92 | 0.80 | 0.70 | 0.69 |
| 2 | bt. D2S151 & D2S142 | 156.65 | 3.07 | 3.19 | 3.29 | 3.30 |
| 2 | D2S142 | 161.26 | 3.34 | 3.46 | 3.56 | 3.57 |
| 2 | bt. D2S142 & D2S2330 | 165.335 | 3.16 | 3.29 | 3.38 | 3.40 |
| 2 | D2S2330 | 169.41 | 2.99 | 3.11 | 3.20 | 3.22 |
| 2 | bt. D2S2330 & D2S364 | 177.81 | 2.39 | 2.51 | 2.61 | 2.62 |
| 2 | bt. D2S117 & D2S2358 | 198.955 | 0.34 | 0.24 | 0.20 | 0.19 |
| 2 | D2S2358 | 203.46 | 0.71 | 0.70 | 0.72 | 0.73 |
| 2 | bt. D2S2358 & D2S2382 | 208.475 | 0.67 | 0.66 | 0.70 | 0.71 |
| 3 | bt. D3S3706 & D3S1597 | 24.445 | 0.72 | 0.76 | 0.80 | 0.80 |
| 3 | D3S1597 | 29.92 | 1.25 | 1.32 | 1.38 | 1.39 |
| 3 | bt. D3S1597 & D3S2338 | 36.01 | 0.82 | 0.87 | 0.91 | 0.92 |
| 4 | bt. D4S403 & D4S2994 | 30.995 | 0.18 | 0.15 | 0.13 | 0.12 |
| 4 | D4S2994 | 36.09 | 0.08 | 0.08 | 0.09 | 0.09 |
| 4 | bt. D4S2994 & D4S391 | 39.84 | 0.07 | 0.08 | 0.09 | 0.09 |
| 6 | D6S462 | 99.01 | 0.78 | 0.68 | 0.45 | 0.41 |
| 6 | bt. D6S462 & D6S1671 | 103.445 | 0.38 | 0.36 | 0.30 | 0.29 |
| 7 | bt. D7S2496 & D7S510 | 53.505 | 0.74 | 0.70 | 0.66 | 0.65 |
| 17 | bt. D17S1791 & D17S799 | 24.94 | 0.27 | 0.32 | 0.36 | 0.37 |
| *Under 0.01 disease allele frequency* | | | | | | |
| 2 | D2S337 | 80.69 | 0.94 | 0.77 | 0.28 | 0.13 |
| 2 | D2S347 | 87.37 | 0.95 | 0.80 | 0.36 | 0.24 |
| 2 | bt. D2S112 & D2S151 | 146.83 | 0.90 | 0.79 | 0.69 | 0.68 |
| 2 | bt. D2S151 & D2S142 | 156.65 | 3.03 | 3.16 | 3.26 | 3.27 |
| 2 | D2S142 | 161.26 | 3.30 | 3.43 | 3.53 | 3.54 |
| 2 | bt. D2S142 & D2S2330 | 165.335 | 3.13 | 3.25 | 3.35 | 3.37 |
| 2 | D2S2330 | 169.41 | 2.95 | 3.07 | 3.17 | 3.19 |
| 2 | bt. D2S2330 & D2S364 | 177.81 | 2.36 | 2.48 | 2.58 | 2.59 |
| 2 | bt. D2S117 & D2S2358 | 198.955 | 0.45 | 0.41 | 0.41 | 0.41 |
| 2 | D2S2358 | 203.46 | 0.83 | 0.85 | 0.89 | 0.89 |
| 2 | bt. D2S2358 & D2S2382 | 208.475 | 0.84 | 0.86 | 0.91 | 0.92 |
| 2 | bt. D2S2382 & D2S126 | 217.31 | 0.39 | 0.41 | 0.46 | 0.46 |
| 3 | bt. D3S3706 & D3S1597 | 24.445 | 0.83 | 0.88 | 0.93 | 0.94 |
| 3 | D3S1597 | 29.92 | 1.34 | 1.41 | 1.48 | 1.48 |
| 3 | bt. D3S1597 & D3S2338 | 36.01 | 0.97 | 1.03 | 1.08 | 1.08 |
| 4 | bt. D4S2935 & D4S403 | 19.93 | 0.05 | 0.03 | 0.03 | 0.03 |
| 4 | bt. D4S403 & D4S2994 | 30.995 | 0.33 | 0.33 | 0.34 | 0.34 |
| 4 | D4S2994 | 36.09 | 0.20 | 0.22 | 0.24 | 0.24 |
| 4 | bt. D4S2994 & D4S391 | 39.84 | 0.18 | 0.20 | 0.22 | 0.23 |
| 4 | D4S391 | 43.59 | 0.11 | 0.12 | 0.14 | 0.14 |
| 6 | D6S462 | 99.01 | 0.75 | 0.66 | 0.43 | 0.39 |
| 6 | bt. D6S462 & D6S1671 | 103.445 | 0.36 | 0.34 | 0.29 | 0.28 |
| 7 | bt. D7S493 & D3S2338 | 40.885 | 0.76 | 0.77 | 0.78 | 0.78 |
| 7 | bt. D7S2496 & D7S2496 | 53.505 | 1.04 | 1.06 | 1.08 | 1.08 |
| 7 | D7S510 | 59.93 | 0.18 | 0.18 | 0.17 | 0.17 |
| 17 | bt. D17S1791 & D17S799 | 24.94 | 0.31 | 0.35 | 0.40 | 0.41 |
| 17 | D17S1791 | 37.485 | 0.20 | 0.13 | 0.04 | 0.02 |

Abbreviations: Chr, chromosome; LOD, log odds score; STR, short tandem repeats; cM, centimorgan; bt., between.
