# Supplementary figures and images for: Expanding the phenotypic spectrum of mutations in LRP2: a novel candidate gene of non-syndromic familial comitant strabismus
Source: J Transl Med. 2021 Dec 6;19:495. doi: 10.1186/s12967-021-03155-z (PMC8647414; doi:10.1186/s12967-021-03155-z)

**Additional file 4**


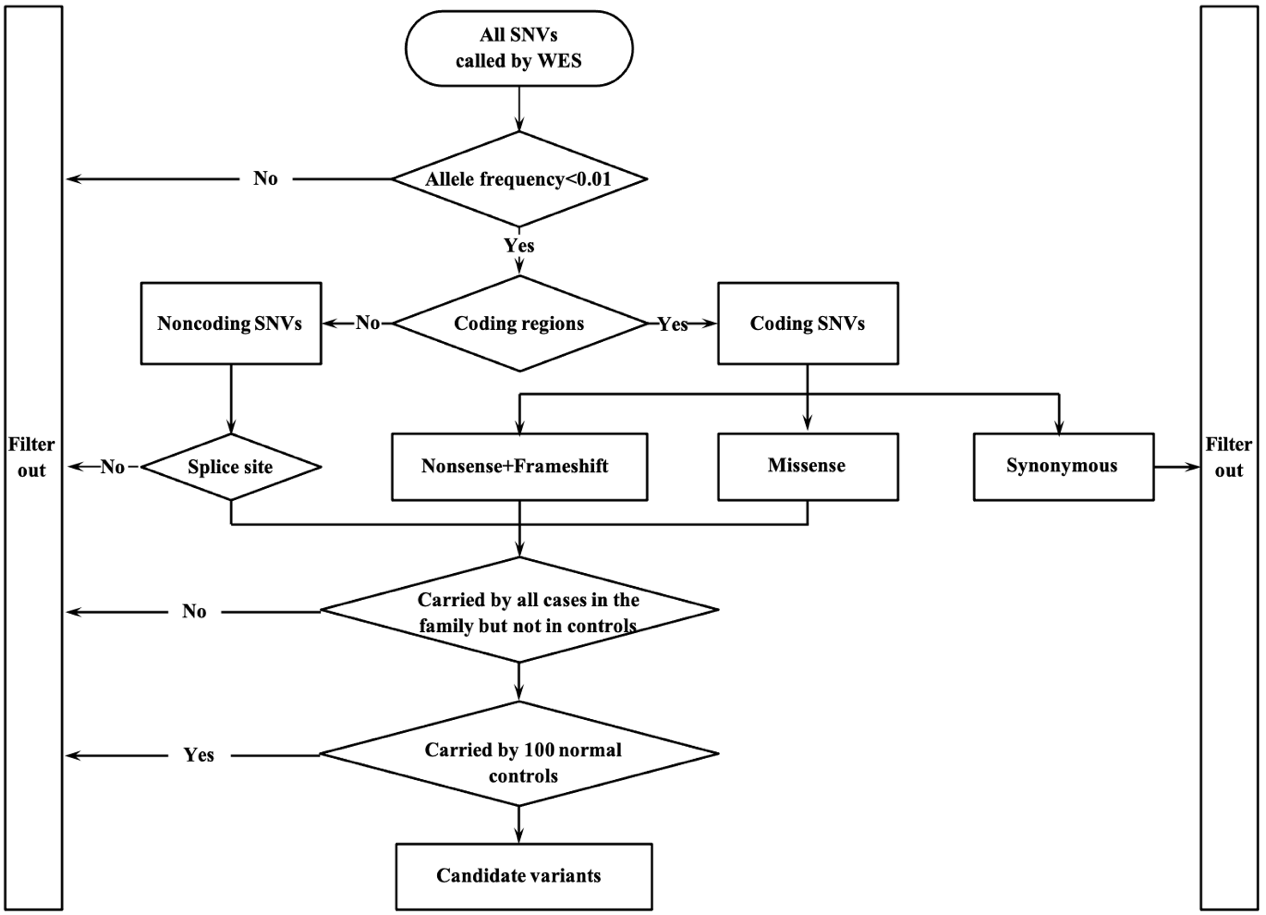


**Figure S2.** Flow chart of variant analyses.

Supplement: Supplementary file 4 — Additional file 4: Figure S2. Flow chart of variant analyses. [file 12967_2021_3155_MOESM4_ESM.docx]
